# Framing as a mechanism to overcome the temptation of bad habits

**DOI:** 10.3389/fnbeh.2025.1683756

**Published:** 2025-10-29

**Authors:** Shuning Wang, John Monterosso

**Affiliations:** ^1^Department of Psychology, University of Southern California, Los Angeles, CA, United States; ^2^Brain and Creativity Institute, University of Southern California, Los Angeles, CA, United States; ^3^Institute for Addiction Science, University of Southern California, Los Angeles, CA, United States

**Keywords:** habits, framing effects, delay discounting, self-control, temptation

## Abstract

Behavioral neuroscience generally conceives of habits as under stimulus-response control, and distinguishes habits from goal-directed behavior based on their insensitivity to outcome value (features of automaticity). However, the everyday meaning of “bad habits” is applied primarily to behaviors that are compelling, in part, because of their anticipated outcome value. In particular, commonly identified bad habits (e.g., overuse of social media, overeating, smoking) are repeated behaviors that yield appealing immediate outcomes, but at a greater longer term cost (“temptations”). We begin by evaluating the role of both automaticity and temptation in the maintenance of bad habits. Next we focus on how framing effects can be used to shift the balance of motivation away from immediate and/or toward delayed outcome value, including a partial summary of what is known about the neural substrates that mediate such shifts. We pay particular attention to the way frames can promote replacing bad habits with good habits through emphasizing the connection between specific choices and general policy preferences.

## Introduction

Self-identified “bad habits” mark a category in which, by the individual’s own evaluation, they consistently do what they ought not (or fail to do what they ought). Psychologists and behavioral neuroscientists have looked to identify the mechanisms that drive these behaviors. Our primary goal here is to develop the case that framing interventions directed at attenuating delay discounting (the decline in present value of rewards with delay to their receipt; Odum, 2011) can be used to reduce bad habits. To this end, we proceed in two parts. First, we characterize essential qualities of bad habits, in the colloquial sense, and differentiate its meaning from the typical usage of the term “habit” within behavioral neuroscience. Whereas insensitivity to current anticipated outcome value is a defining feature of “habits” in behavioral neuroscience, we show that most everyday bad habits are partially maintained by high valuation of more immediate (relative to more delayed) anticipated outcomes. In the second part of this article we show how this characterization can be leveraged to identify interventions that reduce the expression of bad habits through increasing the motivational influence of delayed outcomes. We pay particular attention to the use of framing effects that encourage the individual to conceive singular choices (e.g., whether or not to smoke this cigarette) as holding “self-signaling value” that increases motivation to break bad habits (Ainslie, 1992, 2001; Schelling, 2007).

Within behavioral neuroscience, habits are response dispositions, formed through repetition, and characterized by a set of overlapping attributes including automaticity, inflexibility, cue-dependent execution, and insensitivity to the organism’s current valuation of an anticipated outcome (Drummond and Niv, 2020; Miller et al., 2019; Wood and Rünger, 2016). This last characteristic has been essential to the way habits are identified and studied experimentally. Consider, for example, a rodent trained through food reinforcement to consistently press a lever when it is extended into its cage. Is the lever press a habit – an automatic response to an encountered cue? Or, alternatively, is it goal-directed – based on a conceived (cognitive model) outcome state that the organism values and then acts to reach? To identify which category the behavior falls into, the researcher can intervene to abruptly change the organism’s valuation of the associated outcome (here, its value of the food reinforcer), and then assess whether that change impacts production of the behavior (Balleine and Dickinson, 1998; Bouton, 2021; Dickinson, 1985). For example, if a sunflower seed had been the reinforcer, the animal might be removed from its testing environment and then given sunflower seeds, along with a lithium chloride injection that will make it nauseous. From a single such pairing the sunflower seed will go from highly valued to repulsive (Garcia and Koelling, 1966). If, when returned to the operant training setting, the rodent persists in pressing the lever that had been reinforced with sunflower seeds, then the lever-pressing is considered habitual since its expression is insensitive to current (negative) outcome valuation. Alternatively, if the rodent does not press the lever, that sensitivity to outcome devaluation is taken as indication that the behavior was under goal-directed control.

Work leveraging the above “goal-devaluation procedure” has been used to characterize a transition that occurs during operant training. Early in training with a single instrumental reinforcer, learned behavior is sensitive to the type of changes in value described above. However, after extensive training, the sensitivity to change in current valuation is lost, as evidenced by the persistence of the behavior after goal devaluation (Adams and Dickinson, 1981; Dickinson, 1985). This transition to habit, as defined in behavioral neuroscience (Belin et al., 2009), is accompanied by a shift in primary neural control from the ventral-striatal (including caudate) – associative loop control to the dorsolateral striatal (including putamen) – sensorimotor cortical loop. Human lesion work indicates goal-directed action is dependent on both the vmPFC (O’Doherty, 2011; Reber et al., 2017) and dlPFC (Smittenaar et al., 2013). The medial- to lateral- transition in striatal involvement that occurs with habit formation results in faster and more efficient action, at the cost of reduced flexibility. There is disagreement about whether habits are better functionally characterized as reflecting selection based on previously computed cached value (“model-free” value) (Daw et al., 2006; Dayan and Balleine, 2002) or selection based on a Hebbian process whereby any repeated response comes to be increasingly expressed as a direct function of its response history alone, regardless of past reinforcement magnitude (“value-free”) (Miller et al., 2019). However, on both accounts, the expression of habitual behavior is unaffected by the current valuation of its anticipated outcome.

Goal-directed action can be difficult to distinguish from habits because the degree of sensitivity to current valuation depends on the way decisions are made. For example, an individual confronted with a decision that is similar to one she made last week might decide re-evaluation isn’t worth the mental effort (Shenhav et al., 2017). Instead, particularly early in learning before habits have been formed, she may rely on memory of her past decision, and repeat that behavior (Drummond and Niv, 2020; Lengyel and Dayan, 2008). Like habit control, the heuristic of repeating a past choice diminishes sensitivity to current value. However, unlike habit control, this strategy relies on declarative memory for past episodes. Studies of basic habit-formation have shown the capacity to be normal among amnesic patients (Knowlton et al., 1996) despite their severe impairment in the formation of declarative memories. Whereas declarative memory is highly sensitive to dysfunction of the hippocampus and other structures within the medial temporal lobe (MTL), habit formation is instead highly sensitive to dysfunction of lateral basal ganglia (Mishkin et al., 1984). As discussed below, when a decision is followed as a policy (e.g., a decision to always drink water with meals) true habits may form over time, and untangling the contribution of episodic memory and habits requires experimentation.

## Good habits vs. bad habits in behavioral neuroscience

Habits are generally adaptive. They dramatically reduce the attentional demands required to execute behavior, and so free that limited resource for other tasks. “The more of the details of our daily life we can hand over to the effortless custody of automatism”, as William James wrote, “the more our higher powers of mind will be set free for their own proper work.” (James, 1887, pg. 54). In addition to this processing efficiency advantage, habits can sometimes provide a bulwark against costly shortcomings in decision-making. Someone new to exercising may be prone to forgoing the behavior when they are tired. But someone in the habit of running each morning has context cues that facilitate sticking with the behavior (Wood and Rünger, 2016). Finishing her morning cup of coffee might be a cue that triggers her to next put on her running shoes and get on with her run. She may not even consider the fact that she is not in the mood to run. Indeed those who report the fewest problems with real-life self-control issues are not distinguished by greater “executive function” capacities needed to override automaticity, but instead by a high degree of habitual behavior within domains like health and financial decision-making (Galla and Duckworth, 2015).

However, although on the whole adaptive, particular habits can be dysfunctional. One way this can occur is through divergence between present circumstances and circumstances during habit formation. Habits form in a particular internal and external context, and when the context changes, the habit may become dysfunctional. If for years an individual drives to the same work location each morning, the turns of the route may become habitual. While that allows her to get to work without giving the matter much thought, if her circumstances change, her navigation habit can occasionally cause errors. If she changes jobs she may be prone to mistakenly follow her old route, particularly if her attention is elsewhere while she is driving. Without attention to her current goal, the turns of the former route are selected “automatically.” Her destination has changed, and so her driving route habits will be misaligned with her goals until new driving route habits have had time to form. Another related way goal-habit conflict can occur is through a change in the environment. For an experienced American driver who moves to the UK, a subset of her driving habits will be dysfunctional in the new environment. And here the consequences of that misalignment are far greater because safe driving often requires quickly selecting the correct behavior, and that requisite speed makes driving difficult without functional habits (Petridou et al., 1997).

Both of the above examples of dysfunctional driving habits entail “insensitivity to outcome value.” Neither driving to the wrong location, nor turning into oncoming traffic are attractive outcomes, but both can occur because the mechanism generating these behaviors is not based on a mental model in which expectations are attended to and evaluated prior to selection. They are instead responses elicited by stimulus cues. Both are real-world analogs to errors on executive function tasks such as the Stroop Task in which an automatic response is at odds with goals, and so must be suppressed (Stroop, 1935). We refer to this as the “automaticity challenge” posed by dysfunctional habits. Since overcoming automaticity is dependent on attention, the dysfunctional habit is more likely to be expressed when available attention resources are constrained, such as when responses must be made quickly (Hedge et al., 2019).

## A role for outcome value in a more expansive lay conception of bad habits

However, overcoming “bad habits”, as the term is used outside of behavioral science, is not just a matter of overriding goal-incongruent automaticity. The tendency to drive to one’s former place of work, or for Americans to look the wrong direction for oncoming cars when they move to the UK could be called “bad habits.” But, neither fits with the prototypical “bad habit”, as the category is understood in everyday life, because in addition to their automaticity challenge, prototypical bad habits pose a “temptation challenge”. When people are asked about their own bad habits (YouGov, 2022), highly endorsed behaviors include overeating, smoking, spending too much money, and wasting too much time on social media. These behaviors all include immediate reinforcement and they all hold immediate appeal. Unlike looking the wrong way for oncoming traffic, TikTok videos, hyperpalatable foods, cigarettes (for those who smoke), and shopping sprees are all attractive in the short-term. But they are also, at least for those who identify them as “bad”, viewed to entail more significant long-term costs (in the above, lack of productivity, obesity, cancer, and financial problems respectively). Since more immediate anticipated outcomes tend to be more motivating than temporally distant outcomes (a phenomenon referred to as “delay discounting”), changing bad habits poses a “temptation challenge” that is additional to the automaticity challenge.

The contribution of temptation to prototypical bad habits can explain several characteristics of commonly endorsed bad habits, which would not be expected if it were automaticity alone that made them challenging to break. First, unlike the American driver looking the wrong direction when driving in the UK, or the individual incorrectly reading the word on the Stroop Task, for prototypical “bad habits” the “badness” does not depend on present conditions or goals differing from those during the formation of the habit. The very first time an individual stayed up late binging on TikTok videos she might have viewed her behavior as something she would regret the next day. This implies that for someone for whom the behavior had developed into a habit, even if automaticity could be eliminated it would not guarantee that the bad habit would be broken. Automaticity contributes to the challenge (see below) but for typical bad habits, temptation does as well.

Second, everyday bad habits can be expressed even when attention resources are abundant. The moment the driver on route to her former workplace realizes the mistake, she corrects it without struggle. If the American driver in the UK is at a traffic light and has sufficient time to think through the situation, she is unlikely to look the wrong direction when the light turns green. Goal-incongruent automaticity alone causes errors, but those errors are highly dependent on factors that limit attention such as time pressure (Hommel, 1994; van den Wildenberg et al., 2010) or a distracting secondary task (Pashler, 1994). Imagine if a participant doing a Stroop Task was instructed to think about each response for 10 s before saying it, with a large incentive for accuracy to insure effort. Our intuition is that given those 10 s, achieving the goal of saying the ink color, rather than reading the word would be trivial for healthy participants. Consistent with this, instructing participants to focus more on accuracy than speed greatly reduced Stroop Task errors even as median reaction times per item remained well under a second (Hedge et al., 2019). Individuals struggling to break a bad habit may be helped by an effort to take the time to make each cigarette or each bite a conscious choice. But even when ample attention is given, the bad habits may persist because smoking and eating, like most bad habits, involve a tempting immediate reward.

Third, in seeming contradiction to the way habits are operationally defined in behavioral neuroscience, bad habits are sensitive to anticipated outcome. Consider the case of contingency management (CM) interventions for smoking. While breaking the bad habit of smoking has obvious benefits, those benefits are mostly in the distant future. Moreover, for individuals with nicotine dependence, confidence that they can achieve sustained abstinence is often low (DiClemente et al., 1985), and low confidence is associated with cessation failure (Condiotte and Lichtenstein, 1981; Shiffman et al., 2000). CM interventions for smoking are designed to address these issues by introducing a positive outcome for not smoking that is relatively immediate and achievable. Individuals receiving CM for smoking cessation are given a small daily monetary reward or prize for confirmed abstinence on the previous day (Ledgerwood et al., 2014). Habitual smokers are sensitive to this change in outcome value, as is evident by improved treatment response. For example, a higher percentage of participants trying to quit smoking abstain fully throughout the duration of treatment when a CM intervention is provided (Ledgerwood, 2008; Secades-Villa et al., 2020). CM is also effective for the treatment of other substance use disorders (Dallery and Novak, 2025) as well as for lifestyle changes such as the amount people walk each day (Erath and DiGennaro Reed, 2022; Kurti and Dallery, 2013). Bad habits may be partly driven by automaticity (Neal et al., 2011; Wood and Rünger, 2016), but they are nevertheless responsive to changes in outcome value.

The idea that temptation (the appeal of immediate reward) substantively contributes to the challenge of overcoming bad habits, even after those habits are well-established, appears to be at odds with some important perspectives within psychology and behavioral neuroscience. To some extent the differing perspectives may be explained by the superficial differences in how the term “habit” is used. But we suspect there is some genuine divergence in views about underlying processes as well. Within social psychology, an explicit separation of “bad habits” and “temptation” is, we think, increasingly emphasized. Noting evidence from her lab’s survey work (Quinn et al., 2010), the leading social psychologist on the topic concludes, “Bad habits are not short-term indulgences…habit responses differ from temptations. Habits are more difficult to control, and effortful monitoring and inhibition are the only successful strategies (Wood, 2017, pg. 6). Within behavioral neuroscience, Everitt and Robbins take a similar position. In their influential “maladaptive stimulus-response habit” theory of addiction they argue, “habit learning occurs in parallel with instrumental action-outcome learning but, with extended training, eventually dominates behavioral output. [italics added]” (Everitt and Robbins, 2005, pg. 1485). In other words, when the habit is so strong as to become out-of-control, the represented outcome state is no longer relevant since the behavior is fully controlled by the S-R association (automaticity). The tendency to devalue anticipated delayed costs relative to the anticipated immediate reward of drug use (temptation) no longer matters on this account, since outcome valuation is overpowered by the S-R mechanism. They acknowledge that this conflicts with subjective accounts of drug craving, which center on imagining and desiring (valuing) the drug, but suggest that this subjective experience is epiphenomenal and misleading, and that craving may arise, “*post hoc* as a rationalization of the “out-of control” habitual behavior rather than being the driving influence” (Everitt and Robbins, 2005, pg. 1485). Arguments against the view that S-R dominance accounts for typical strong bad habits are noted above, including the fact that addictive habits are sensitive to changes in outcome value (Dallery and Novak, 2025). For a broader set of arguments contradicting the automaticity account of addiction, see Heyman (2009).

Automaticity alone is unlikely to explain the challenge people experience when they try to break their bad habits. The smoker might sometimes light up mindlessly (automatically), but when she makes a serious quit attempt, the topic has her attention. Unlike the individual driving to the wrong office out of habit, the relapsing smoker on her way to buy cigarettes does not abort her mission and return home the moment she realizes where she is going. Nevertheless, automaticity may strongly contribute to the recalcitrance of bad habits by biasing the output of goal-directed (valuation-based) action. While the set of actions possible at any moment is limitless, the set of actions actually evaluated is necessarily extremely small given the demands evaluation places on attention. The S-R process unfolds rapidly, and so can provide the first action-outcome possibility to be evaluated. Even when behavior is selected through outcome valuation, automaticity may bias value-based choice by providing the first (and often only) outcome to be evaluated. If I have repeatedly smoked after each meal, the empty plate brings the idea of smoking to mind. That consideration given to the habitual response strongly biases action selection, even as the execution of the behavior retains sensitivity to valuation.

In addition to biasing what behaviors get evaluated, associative pairings acquired through learning can entrench bad habits by creating surges in motivation (Berridge and Robinson, 1998). The same stimulus that triggers the bad habit response through automaticity (e.g., for the smoker above, the conclusion of the meal) also functions as a Pavlovian cue with a reinforcement history linking it to nicotine. There is extensive evidence that encountering a Pavlovian cue predictive of reward increases expression of instrumental responding for the same reward, a phenomenon called “specific Pavlovian-instrumental transfer” (PIT) (Corbit and Balleine, 2005). Specific PIT depends on circuits linking the basolateral amygdala and nucleus accumbens shell (Corbit and Balleine, 2005, 2011). Rather than simply triggering behavior, such cues may function by increasing the perceived efficacy of the associated action—i.e., the likelihood that it will successfully yield the predicted reward. Through this transfer, Pavlovian learning closely linked to the acquisition of automaticity may amplify “wanting” for an outcome (Berridge and Robinson, 2003), and so enhance the temptation challenge associated with breaking bad habits.

## Delay discounting as a prominent contributor to the maintenance of bad habits

As noted above, delay discounting refers to the reduction in valuation of expected outcomes the more they are delayed. While not the only driver of temptation (Loewenstein, 1996; Metcalfe and Mischel, 1999), the asymmetry in the timing of desirable vs. undesirable consequences contributes to most bad habits. In particular, behaviors characterized as “bad habits” typically provide something attractive right away, but entail some significant cost in the long-term. Consider how different the situation would be if the outcome timing of bad habits were reversed. Imagine the case of the individual with a bad habit of staying up late binging on TikTok, but instead of a series of immediate small rewards followed by feeling a worse the next day, selecting TikTok videos made her feel immediately worse, and the entertaining videos did not play until the next morning. Or for the individual trying to break a heroin habit, imagine the feelings of shame and social isolation occurred moments after the needle pierced the skin, but the high didn’t arrive until the next day. We think it is self-evident that flipping the timing would make these habits easier to break. And in cases in which the bad habit is inaction (e.g., procrastination), it is also true that persisting in the habit (e.g., not doing the unpleasant task that needs to be done) holds immediate appeal but delayed costs. Given its role in perpetuating bad habits, interventions that reduce delay discounting, either generally or in a way that is linked to the outcomes of a particular habit, have the potential to facilitate breaking bad habits.

## How is delay discounting studied in the lab?

Within psychology and neuroscience, work on delay discounting relies heavily on a particular type of experimental model called the “delay discounting task.” The variants of the task used with humans evolved from methods developed by operant psychologists studying reinforcement in rats and pigeons (Ainslie, 1975; Herrnstein, 1970; Mazur and Logue, 1978; Rachlin and Green, 1972). In a typical delay discounting experiment, animals are trained on two responses, one of which is reinforced by the smaller but sooner (SS) reward, and the other of which is reinforced by the larger but later (LL) reward. For example, in a study using two different retractable levers as operants, pressing one lever could be reinforced with 1 food pellet delivered immediately (the SS), and pressing the other lever by 2 pellets that are delivered after a 3-s delay (the LL). Because only one lever is available on many trials, the animal learns about each. However, intermixed within single-alternative trials are critical “choice trials” in which both levers are extended into the chamber until either is pressed. With both options available, the response made can be treated as an indication of which reinforcer is greater. Does the organism choose 1 immediate pellet or 2 pellets delayed by 3-s? And by parametrically varying the amounts and delays used in different conditions of the experiments, an overall “delay discount function” can be derived to characterize how reinforcement declines with delay.

The basic logic of the animal operant choice studies was subsequently adapted for use with people. Occasionally the delayed rewards or punishments in human research have been administered in real-time (Logue and King, 1991; McClure et al., 2007; Reynolds and Schiffbauer, 2004), but much more often the adaptation of the task in humans presents alternative rewards (often hypothetical or real money) in linguistic/numerical abstraction, with delays that are too long to experience in the lab. Participants are asked questions like, “Would you prefer $10 in 1 day or $15 in 25 days?” Either through sampling a wide range of preset alternative pairs (Kirby et al., 1999) or through adaptive questioning, participant responses allow the researcher to estimate the impact that delay has on value (Ahn et al., 2020; Cavagnaro et al., 2016; Mahalingam et al., 2018). A variety of approaches can be used to characterize data from these procedures, but as with data from operant experiments, a common approach is to fit data to a delay discount function characterizing the impact of delay on value (Rachlin, 2006). There is disagreement regarding the best specification of the discount function, and whether a single or multiple functions should be used to fit data (Andersen et al., 2014; Harrison et al., 2010), but research in psychology has relied primarily on some variant of the “hyperbolic discount function” (Kirby and Maraković, 1995). Hyperbolic discounting makes value inversely proportional to delay, and unlike exponential discounting, it is at odds with neoclassical “rational actor” models since hyperbolic discounting implies preference reversals based on the passage of time (Ainslie, 1975; Laibson, 1997; Monterosso and Ainslie, 1999). Alternative approaches to fitting discount functions include characterizing responses using a model-free “area-under-the-curve” score (Borges et al., 2016; Myerson et al., 2001), or as the product of which heuristics participants adopt (Marzilli Ericson et al., 2015).

## The delay discounting *task* is not the delay discounting *topic*

Since delay discounting contributes to the temptation of bad habits, framing effects that reduce delay discounting may be beneficial for overcoming them. However, before considering research on this topic, it is important to emphasize that the delay discounting task described above is an idealized model. As with any model, its specifications should be considered when drawing inferences. In the delay discounting task, participants are asked to select between pairs of alternatives, whereas the set of options in the real world is typically open. In the task, alternatives are specified with precise information about reward magnitude and reward timing, but expectations in the real world are rarely so well-specified, especially with regard to outcome timing. Consider the decision to have a snack in the afternoon – how and when does that decision impact the individual? There is the pleasure eating, which has a temporal profile that unfolds over seconds, or perhaps minutes. There are the ways the food impacts the body in the subsequent hours (less hunger pangs, but perhaps more sluggishness). There is the effect the consumption will have on the enjoyment of dinner later that evening. There are potential effects the snack could have on health and appearance (effects that are small but temporally expansive). There are potential knock-on effects that the snack could have on future food decisions (including contributing to the formation of a snacking habit that is difficult to break). These contingent outcomes are not well specified in either their magnitude or their timing. And the situation is arguably even more complex with respect to delay in interpersonal decisions, where how we behave can change the way others subsequently behave toward us, and what we experience them as feeling about us. While the delay discounting lab task has been validated for some purposes (see below), it should not be presumed to capture all that is relevant to real-world response to delayed outcomes.

## The delay discounting task appears to be a useful model

The above limitations notwithstanding, the delay discounting task continues to be widely used in behavioral science, including behavioral neuroscience, because it performs well in important ways (Amlung et al., 2019; Bickel, 2015; Lempert et al., 2019). Individual differences on the delay discounting task have high test-retest reliability across the span of up to at least a year (Kirby, 2009; Kräplin et al., 2016; Simpson and Vuchinich, 2000). Behavioral genetic studies indicate that the delay discounting task has moderate to high heritability (Anokhin et al., 2011, 2015), suggesting an opportunity to use the construct of steep delay discounting as an endophenotype relevant to psychiatric conditions (Bickel, 2015; Lempert et al., 2019). Moreover, genome-wide association studies have identified particular genes that explain some individual differences in task performance (Cupertino et al., 2023; Sanchez-Roige et al., 2018).

But is there evidence that steep discounting on the delay discounting task marks a disposition to the formation and persistence of bad habits? Is it the case that an individual who greatly (relative to other people) prioritizes getting rewards sooner is more prone to bad habits? This is a difficult question to answer definitively because work in the area necessarily relies on correlational data. However, there is extensive evidence indicating greater than typical delay discounting among populations with drug addiction (Amlung et al., 2017; MacKillop et al., 2011; Weinsztok et al., 2021) and some other real-world behaviors that could be considered “bad habits” such as excessive shopping, gambling, and internet use, (Alessi and Petry, 2003; Cheng et al., 2021; Williams, 2012). Moreover, the idea that steep delay discounting plays a causal role in bad habits is supported by longitudinal evidence that adolescents who discount more steeply on the delay discounting task are more likely to subsequently develop smoking addiction (Audrain-McGovern and Benowitz, 2011; Felton et al., 2020). Interestingly, the particular genes that have been identified as explaining variance in delay discounting overlap substantially with genes linked to behavioral problems associated with steep delay discounting, such as smoking and obesity (Cupertino et al., 2023; Sanchez-Roige et al., 2018).

## Neuroimaging work on delay discounting

Neuroimaging work related to the delay discounting topic has relied heavily (though not exclusively) on the delay discounting task. The earliest work on the topic married analysis of neuroimaging data acquired while participants completed a delay discounting task to a particular discount function commonly referred to as either, “quasi-hyperbolic discounting” (Laibson, 1997), or “beta-delta discounting.” In the model, all delayed periods are discounted categorically using a multiplicative “beta” parameter that is less than 1, and additionally by an exponential “delta” parameter applied to delay, with the latter used to fit delay’s continuous impact on value. In the first reported study examining brain activity while participants completed a delay discounting task, the researchers modeled participants’ behavior using the beta-delta function and then used each of the fit parameters (beta and delta) as predictors of brain activity. The findings from that groundbreaking work provided initial suggestion that choices in a delay discounting task, and perhaps more generally in the type of situations in which temptation can lead to bad habits, reflect the outcome of a dual-system neural architecture in which brain “beta regions” highly sensitive to immediate rewards (especially the limbic system) compete with “delta regions” (including the fronto-parietal executive function network) that weigh the future more rationally (Bickel and Yi, 2008; McClure et al., 2004, 2007). If sustained, this conclusion could provide a brain basis for dual-system accounts such as the “planner and doer” model proposed by Thaler and Shefrin. According to that model, response to temptation can be modeled as, “two sets of coexisting and mutually inconsistent preferences: one concerned with the long run, and the other with the short run” (Shefrin and Thaler, 1992). However, there is substantial evidence that contradicts the beta-delta system competition account of delay discount task performance (Kable and Glimcher, 2007; Koban et al., 2023; Monterosso and Luo, 2010). In a study that looked at value tracking in limbic “beta regions”, Kable and Glimcher observed no evidence that these regions were hypersensitive (that is, more sensitive than would be expected based on behavior) to immediate reward (Kable and Glimcher, 2007). And in work in our lab we observed no evidence that value tracking in delta regions was hypersensitive (again, relative to behavior) to delayed rewards (Luo et al., 2009, 2010).

Another theoretical approach that is relevant to the temptation component of bad habits emphasizes the link between delay discounting and prospection or, “mental time travel” (Tulving, 1985). In the 1930’s the psychologist Karl Muenzinger noticed his rats running in a T-Maze would pause at the junction, sometimes leaning one way and then the other before proceeding down one arm of the maze (Muenzinger, 1938). It appeared to Muenzinger that the rats were evaluating possibilities by imagining each before acting – a process Muenzinger and Edward Tolman (both foundational figures in early animal cognition research) referred to as “vicarious trial-and-error.”(Muenzinger, 1938; Tolman, 1939). When the field of psychology entered the era of behaviorism, such inferences about mental life were treated by most of psychology as unscientific (Skinner, 1965). However, many decades later, David Redish and colleagues would lend neuroscientific support for Muenzinger and Tolman’s inference by decoding the firing of neuronal “place cells” in the hippocampus during that same pre-decisional pausing behavior in their rats (Redish, 1999, 2016). The sequential activity of place cells corresponding to one side and then the other fit with the animal cognitions that had long ago been inferred, and the location of the place cells that fired immediately prior to decisions predicted the rat’s subsequent direction of travel.

Of course, the mental time travel of the rodent is quite limited relative to the human. People spend much of their time “mind wandering”, thinking about the future and replaying the past (Killingsworth and Gilbert, 2010). Both of these activities require a shift in perspective beyond the immediate environment, both require harvesting of memory to generate counterfactual stimuli, and both require keeping track of the distinction between the counterfactual and the present (Buckner and Carroll, 2007; Kurczek et al., 2015). Not surprisingly, the brain regions implicated in each are highly overlapping (Addis et al., 2007; Hassabis et al., 2007; Schacter et al., 2007; Schacter and Madore, 2016; Tulving, 1985) and include the vmPFC, the medial temporal lobes (including hippocampus and parahippocampal gyrus), the precuneus, and the posterior cingulate cortex (Addis et al., 2007; Botzung et al., 2008; D’Argembeau et al., 2008; Stawarczyk and D’Argembeau, 2015). The capacity to mentally simulate the future provides a mechanism by which temporally distant expected outcomes can generate the level of motivation necessary to overcome the immediate temptation of bad habits (Bechara, 2004).

Consistent with its hypothesized role of mental time travel in farsighted choice, a study by Peters and Büchel (2010) interjected episodic future imagery “tags” (reminders about events in the participant’s personal anticipated future like an upcoming appointment to get a haircut). The tags, which were not intrinsically linked to the delay discounting task, were hypothesized to prime engagement of prospection. Consistent with their hypothesis, the researchers observed a shift toward less delay discounting in the prospection-prime condition, accompanied by greater recruitment within the episodic imagery network and greater coupling of elements in that network to the anterior cingulate cortex (ACC). In the case of overcoming bad habits, since long term outcomes generally favor their discontinuation, interventions promoting episodic imagery/ future thinking, could facilitate overcoming bad habits. Importantly, even when individuals are not directly engaged in mental time travel, they may behave in ways that are informed by past instances in which they were. Fear related to the mental-time-travel-based image of being in a hospital after a coronary event might be instrumental in motivating an individual to break their habit of buying beef, but with repetition, that image need not be brought to mind every trip to the grocery store.

Another important approach to modeling delay discounting task performance emphasizes the role played by the allocation of attention. The alternatives on delay discounting tasks are characterized by two distinct attributes: reward magnitude and temporal delay. And since each alternative is better on one attribute but worse on the other, preference depends importantly on how attention is allocated between these attributes (Amasino et al., 2019; Cao et al., 2021). That attention shapes preference construction is well-supported in prior research (Orquin and Mueller Loose, 2013; Milosavljevic et al., 2012; Towal et al., 2013), and neuroscience evidence further shows that attention allocation moderates the directional influence of fronto-parietal activity on delay discounting (Koban et al., 2023). Computationally, this attentional perspective is formalized in models such as the Comparison with Goal States Model (CGSM; Suri and Paap, 2024), which proposes that attention amplifies activation of options whose representations are more similar to current goal states. Through this mechanism, CGSM accounts for how less-preferred but goal-consistent options can come to dominate the decision process. Interestingly, simulations also show that the temporal alignment of attention with distal goals can shift the form of the discount function, resulting in either hyperbolic or exponential functional form.

## Delay discounting and framing effects

In an important set of studies, Ebert and Prelec demonstrated that, in addition to the general hyposensitivity to delay length, the impact of delay is “fragile.” It is substantially altered through superficial context manipulations (Ebert and Prelec, 2007). The discounting participants exhibit on the delay discounting task can be manipulated by a variety of methods including the interjection of incidental emotional primes (Luo et al., 2012) or factors that affect the relative salience of amount vs. delay information (Cao et al., 2021). In studies using eye-tracking to estimate participants’ attention to attributes, salience manipulations affect visual fixation times and alter delay discounting in a way consistent with enhanced influence of the salience-enhanced attribute on decisions (Milosavljevic et al., 2012; Shimojo et al., 2003; Towal et al., 2013). In particular, there is evidence that the duration of attention to an attribute affects its weighting during decision-making (Orquin and Mueller Loose, 2013).

A primary claim of this paper is that “framing effects” can be leveraged to disrupt bad habits by increasing temporally farsighted behavior. Framing effects refer to cases in which the way contingency information is presented impacts participants’ valuation of alternatives. Tversky and Kahneman (1981) analogized decision problems to the challenge of visually perceiving the external world accurately, despite the fact that the same scene can be framed from different angles. Framing effects are commonly driven by the reference point used when presenting information. In perhaps the most famous example of a framing effect, participants were asked to decide between two treatment options for a group of 600 people with a sometimes fatal disease. In the “gain frame” condition, intervention alternatives were described as A) certainty of saving 200 people vs. B) a 1/3 chance of saving all 600 people, but a 2/3 chance of saving nobody. Notice that the implied reference point in this condition is everybody dying. In the “loss frame”, the objectively identical outcomes were instead described as A) certainty 400 people will die vs. B) a 1/3 chance nobody will die, but a 2/3 everybody will die. In this second frame, the implied reference point is everybody living. The more risky option was considerably more attractive to participants in the latter “loss frame” than in the “gain frame” (Tversky and Kahneman, 1981). More generally, people are more risk-seeking in their choices, whatever the domain, when the reference point is such that it frames outcomes as losses rather than gains (Kahneman and Tversky, 1984).

Within psychology and neuroscience delay discounting has been shown to be sensitive to framing effects (DeHart and Odum, 2015; Rung and Madden, 2018). Perhaps the most common approach is to examine discounting in a context in which either the SS or the LL is established as the default (making it the natural reference point). For example, in one condition of a study examining framing effects in intertemporal decisions, participants first indicated what they would pay for a tech device that they would receive after some specified delay, and were subsequently asked how much the price would have to be reduced for them to agree to have delivery further delayed by some additional amount of time (the “slow-down” condition). Other participants were asked first a similar question followed by how much extra they would pay to have delivery expedited by some amount of time (the “speed-up” condition). The inferred discounting based on the slow-down condition was substantially greater than discounting inferred based on responses during the speed-up condition (Loewenstein, 1988). Similar results are observed across a wide range of time horizons (Malkoc and Zauberman, 2006). Prospect Theory provides a plausible explanation for these framing effects. The status-quo established in the “slow-down” makes the delay difference between the alternatives a loss and the price difference a gain, while the opposite is true for the speed-up framing. The fact that participants showed greater sensitivity to delay (i.e., steeper discounting) in the slow-down condition is, therefore, consistent with extensive evidence from Prospect Theory that the value function is steeper in the loss domain than gain domain (Tversky and Kahneman, 1981). Neuroimaging studies have identified neural correlates of this asymmetry. Sun et al. (2022) found that the framing effect recruits domain-specific neural circuits: in the gain domain, it is associated with right amygdala activation and enhanced amygdala–vmPFC connectivity; in the loss domain, it involves greater putamen activation and modulated connectivity with dmPFC.

How might reference-dependent framing impact efforts to break bad habits? By definition, habits have become the individual’s default response to a given situation. Forgoing the immediate reward of a bad habit, such as snacking before bed, might, therefore, tend to take a loss-frame (as opposed to forgone gain), whereas any associated improvements in expected long-term outcomes would take on a gain frame. Since utility functions are steeper in the loss than gain domain (Kahneman and Tversky, 1979), that default framing would be expected to add to the challenge of breaking bad habits, even on those occasions in which there is engagement in value-based decision-making. Situations or manipulations that alter the perceived *status quo* may facilitate breaking habits by reducing the motivation advantage linked to their status as the behavioral default. Emphasizing a clean slate, “today is the first day of the rest of your life.” or capitalizing on a transition such as moving (Verplanken and Wood, 2006) or just the start of a new year (Oscarsson et al., 2020) can disrupt the reference-dependence advantage associated with continuation of habits. Consistent with this, a framing manipulation designed to get participants to view a period as a “fresh start” significantly impacted food choice in line with disruption of bad habits (specifically, increased preference for healthy options) (Yu et al., 2023). Moreover, Price and colleagues observed that individuals who were generally quick to adopt a fresh-start mindset, as indicated by agreeing with statements like, “An individual can let go of the past and start anew.” invested more in breaking bad habits (Price et al., 2018). Framing effects may also contribute to the aforementioned association between optimism about change (high self–efficacy) and success breaking bad habits. When an individual views themselves as having broken a bad habit (e.g., believes she has quit smoking for good) the *status quo* changes. For someone engaged in smoking cessation, it is plausible that the more she has come to see herself as a former smoker, the more she would experience smoking as losing something (in particular, losing her valued identity as a former smoker). The greater the self-efficacy, the sooner the bad habit may come to take on a loss frame, thereby reducing the bad habit’s appeal (though high confidence may entail other risks, discussed below). An individual with low self-efficacy may be slow to believe she has really broken a bad habit for good, and so may be slow to benefit from a shift to a loss frame when considering engaging in the behavior.

Finally, there are many studies that demonstrate reductions in discounting through manipulations that enhance the vividness with which participants imagine future events. Some of these involve interventions that do not fit the above characterization of what a framing effect is. For example, the vividness of imagining one’s distant future self through a third-person perspective can decrease delay discounting (Macrae et al., 2017) as can viewing a computer-aged picture of oneself (Ersner-Hershfield et al., 2009). Although these interventions are not “framing effects”, their impact may be related to the “explicit date” framing effect on delay discounting. It has been repeatedly observed that participants engage in less delay discounting when the same information is conveyed as actual dates rather than in interval form (Jiang and Dai, 2021; Keidel et al., 2024; LeBoeuf, 2006; Read et al., 2005). While the cause or causes of this well replicated finding remain unsettled, it is plausible that specifying a date makes the delayed outcome more salient, thereby evoking more prospective imagery. This possibility is consistent with observed elevated activity within the precuneus/ posterior cingulate cortex and angular gyrus (regions linked with episodic imagery (Schacter et al., 2017) when dates are used rather than delay intervals (Keidel et al., 2024).

## Framing effects and *policy* over *particulars* in breaking bad habits

Consider the habitual smoker who is worried about her health, but who currently craves a cigarette. In deciding whether to light up, she faces a decision that pits immediate reward/relief against the perceived harm linked to that cigarette. But what is the harm of one more cigarette? Approached singularly, the literal expected incremental health impact of one more cigarette is distant and small (whereas the reward of smoking is immediate and salient). Similarly, the literal impact the single next rich dessert will have on the habitual overeater’s weight, or the single next unnecessary item off Amazon on the habitual spender’s financial future are both quite small. But, critically, we don’t just hold preferences for singular choices. We abstract categories of behavior and hold policy-level preferences about those categories, such as a preference to be a non-smoker. For the two-thirds of smokers who want to quit but who continue to smoke the next cigarette, there is a tension between immediate single preferences and policy preferences. Among the many suggestions made regarding how policy-level preferences sometimes win out over bad habits, we will consider two. First, we consider the idea that policy prevails over bad habits through heightened salience of a more expansive conception of the situation (promotion of a “forest-not-the-trees” perspective). Second, we consider the idea that policy prevails over bad habits through an emergent sense that single immediate choices hold high significance for achieving policy-level preferences – a phenomenon the behavioral scientist George Ainslie termed, “bundling” (Ainslie, 1975). We will consider these related ideas in turn, including how framing effects could be used to promote each.

## Reducing temptation by enhancing attention to the more abstract construals (seeing the forest-not-the-trees)

The immediate particular -vs.-policy preference conceptualization of temptation suggests a possible way that framing can be used to promote breaking of bad habits. Breaking bad habits may be facilitated by framing choices in a way that increases the relative salience of the policy level. Construal Level Theory (CLT) posits that psychological distance (including temporal distance) influences how individuals mentally represent objects and events. When an anticipated event is distant, representation tends to be abstract, whereas when an event is near in time, representation is concrete (Trope and Liberman, 2010). From a distance, the plan to host your niece next month may be construed broadly as an opportunity to build bonds with family. When the same event is tomorrow, the specific details become more salient, perhaps including the effort of preparing and the ways the visit will disrupt an already busy schedule (Gilbert, 2006). Importantly, while temporal distance impacts construal level, other factors do as well. For example, Fujita et al. (2006) manipulated construal level by requiring participants to think about either “how” particular actions were accomplished (priming lower level construals) or about “why” particular actions were taken (priming higher level construals). Participants assigned to the higher level construal condition subsequently exhibited less delay discounting. Moreover, as shown in an interesting set of studies by Fujita and colleagues (Fujita and Han, 2009; Carnevale et al., 2015) the experimental induction of high-level construals tends to bias action away from immediate temptation (e.g., reduced positive implicit associations of candy bars relative to apples). In addition to promoting high-level construals through framing that primes more abstraction, there is evidence that presenting the same contingencies in gain (rather than loss) frames promoted higher level construals (Chang et al., 2015). In work that directly probed construal-level through an experimental “how” vs. “why” manipulation, the medial wall of the anterior prefrontal cortex was implicated in high-level construals (Stillman et al., 2017). Other more recent work additionally suggests high-level construals are marked by increased integration across brain networks, as indexed by global efficiency (Stillman et al., 2020).

## Breaking bad habits through intrapersonal bargaining

Construal Level Theory provides a compelling account of factors that contribute to identifying dysfunctional patterns of behavior through adoption of an expansion of regulatory scope (Fujita et al., 2025). An individual who habitually looks at his phone when spending time with his child may see that behavior in a negative light when he adopts a high-level construal, and that may promote breaking the bad habit. But CLT has less to say about the effort to break a well-recognized and longstanding bad habit. The typical individual who identifies smoking as a bad habit is well-aware of its harmful health consequences. Her policy-level preference to be a nonsmoker may even be a strong one – maybe she would pay money if doing so could somehow have the effect of ensuring she quits for good. At the same time, her preference for the next cigarette may also be strong. While it is true that people may sometimes act habitually in ways that they do not realize undermines their interests, the behaviors people think of as “bad habits” are necessarily known to them. Bad habits are cases in which insight alone has not resolved the clash between policy and immediate single choice preferences.

Ainslie’s intrapersonal bargaining account characterizes how the will to break a bad habit can emerge through cognitions that raise the stakes of each temptation. As a starting point, notice that for bad habits, the direct tension between the single particular preference and the policy preference is actually slight. It is only the immediate cigarette for which the particular and policy preferences pull in opposite directions. A natural response is to “carve out” now, or today, as an exception to the policy. Smoke today, quit tomorrow. Indulge in fast food today, start a healthy diet tomorrow. The appeal of “just this last one”, is obvious, but so too is its flaw, since each tomorrow turns into the new “today”, and plans to break the bad habit are always a day away. Critically, if for the past 5 years I continually planned to break my smoking habit “soon”, my current identical plan to do the same is no longer credible. That loss of credibility is a loss of utility, since the present value of the “nonsmoker” policy preference depends on believing that it, along with its associated expectations like better health, will come to pass. According to Ainslie’s “intrapersonal bargaining” account, that lack of credibility of, “just this last one” is essential for breaking a bad habit (Ainslie, 1975, 1992, 2001). For the individual who is uncertain about achieving her policy preference to be a nonsmoker, forgoing the immediate cigarette may come to take on added significance as necessary for maintaining credibility of the policy. The literal harm of one more cigarette is tiny and distant, but if someone has resolved to never smoke again, smoking that one cigarette may be experienced as more costly than its literal impact. It may carry signaling value to the individual that changes their expectations about achieving their policy preference to be a nonsmoker. In such a case, cessation depends less on the expected literal impact of the particular cigarette, and more on expectations that have become coupled to each individual choice. Viewing something more general than the literal effect of one more cigarette to be “bundled” with a singular decision is a mechanism that can sometimes facilitate breaking a bad habit (Ainslie, 1992; Prelec and Bodner, 2003; Rachlin, 2004; Bénabou and Tirole, 2004).

The above idea of “bundling” is less a strategy that individuals need to be introduced to, than it is a characterization of what people spontaneously do when they struggle to break a bad habit. This can perhaps be made evident through a thought experiment. Imagine a smoker who wants to quit but who is currently strongly craving a cigarette. Her decision of whether to smoke or not is on a knife’s edge. And imagine in that moment she becomes suddenly certain that from tomorrow on she is destined to continue smoking her pack a day. Would there be any point to resisting her craving? Or alternatively, imagine she instead became suddenly certain of the opposite– that from tomorrow on, she was destined to never smoke again. Would she resist the current craving if she knew it really would be just this last one? Our intuition is that in both cases in which the future is understood to be already set, the individual would give in to temptation. And there is some empirical support for this, since both underconfidence (Marlatt and Donovan, 2005; May et al., 2003) and overconfidence (Goodie, 2005; Zhang et al., 2016) are associated with subsequent failure of self-control in domains like drug addiction and problem gambling. The observation that fixing the future undermines breaking bad habits, according to Ainslie (1992, 2001), see also, Schelling, 2007 suggests that ordinarily, resisting a single particular temptation is enabled by the sense that it holds significance for the future.

The theorized cognitive and neural processes supporting the above intrapersonal bargaining are difficult to study empirically since the proposed mechanism involves an endogenous process of internal feedback between expectations and preference (expected outcomes inform preferences, and preferences inform expected outcomes). Empirical work on the topic has generally attempted to model components of the process. Most directly, several studies have shown that when selections are literally bundled such that whatever choice is made in the present determines the same outcome repeatedly over time, both nonhuman (Ainslie and Monterosso, 2003; Stein et al., 2013) and human (Hofmeyr et al., 2011; Kirby and Guastello, 2001) participants make more farsighted choices. For example, when choosing between an SS and LL food reward that would occur five times over 5 weeks, during the first week participants expressed greater preference for the LL option when the experimental condition specified that the choice would determine the same outcome for all 5 weeks (relative to a condition in which each week was independent). Interestingly, in another study cigarette smokers were significantly more patient when an association between present and future choices was merely suggested, “…What somebody chooses 1 week is often what they go on choosing in later weeks, but you’ll be completely free to choose between these two options every 2 weeks.”(Hofmeyr et al., 2011, pg. 404).

An alternative approach to empirically investigating intrapersonal bargaining is to use interpersonal bargaining in an iterated prisoners’ dilemma as a model. Just as restraint can emerge because the individual comes to view their own future behavior (e.g., being a smoker vs. nonsmoker) as linked to her present choice (Ainslie, 1992), cooperation can emerge in an iterated prisoners’ dilemma when the individual views their counterpart’s cooperation as dependent on their own (Axelrod, 1980). Using this approach a “lapse” was modeled as the introduction of feedback given to players engaged in mutual cooperation that falsely indicated defection. A single round of false-feedback that one’s counterpart defected led to a lasting increase in defection rates, with cooperation rates not recovering for many rounds (Monterosso et al., 2002). This contrasted with false-feedback of cooperation presented to players engaged in mutual defection, which had no significant impact beyond the single next round of play. This pattern is suggestive of an intrapersonal analog to the “abstinence violation effect” (Curry et al., 1987) discussed below.

The intrapersonal bargaining perspective on breaking bad habits suggests a potential benefit of framing individual choices in ways that imply their connection to a larger category. For example, in 12-step programs, adherents are explicitly encouraged to frame future choices as bundled together with the immediate present temptation. What might look like a particular decision to have one drink is not really about “just this one” because, according to 12-step doctrine, once someone with alcoholism starts they cannot stop (“One drink is too many and a thousand not enough”, as writer Brendan Behan put it, based on his own struggles). There may be a downside to framing a single decision as not really singular, including its potential link to the “abstinence violation effect” (Monterosso and Ainslie, 2007) whereby even small lapses lead to periods of full-blown relapse due to negative emotions and loss of self-efficacy (Curry et al., 1987; Marlatt and Donovan, 2005). But it is noteworthy that the 12-step approach is successful (relative to other interventions) at promoting abstinence in the context of drug addiction (Kelly et al., 2020), and has also been successfully applied to behavioral addictions (Schuler et al., 2016).

At present, there is little that can be said about the behavioral neuroscience of intrapersonal bargaining, beyond the general speculation that reasoning about the precedent value of present temptations is likely to depend strongly on both episodic memory (which is critical for viewing the “just this last one” plan as not credible) and future thinking (which is critical in constructing plans about future behavior linked to the present choice). Since both types of episodic imagery are strongly dependent on the medio-temporal lobe network (Atance and O’Neill, 2001; Brunette et al., 2019; Schacter and Madore, 2016), its functioning is likely critical to overcoming bad habits through intrapersonal bargaining.

## Conclusion

The “bad habits” people recognize in their own lives are hard to break because of both automaticity and immediate temptation. Automaticity implies that the dysfunctional behavior is repeatedly chosen, typically with little contemplation. But even when the individual does contemplate the alternatives, there is something immediately rewarding about most bad habits. The classification implies a tension between policy preferences and preference for specific immediate rewards (e.g., a preference to be a nonsmoker, but also to smoke the next cigarette). In this examination of how framing effects can disrupt bad habits, we focused primarily on ways framing can reduce temptation. This included framing interventions that change the reference delay, that directly make the future feel closer, that increase the “regulatory scope” of considerations, and that promote breaking bad habits by enhancing the degree to which individuals experience their current decision as holding implications beyond what is literally at stake. Importantly, none of these interventions are mutually exclusive with one another. Indeed there may be potential synergies between proposed mechanisms. A promising possibility, we think, is that intrapersonal bargaining, which relies on conceiving specific choices as having big-picture implications, may be facilitated by framing primes that promote higher construal levels. The increased salience of an abstract perspective may increase the perceived link between particular choices and achieving policy preferences. We think assessing this possibility in future research on breaking bad habits is a promising opportunity.
